# Addition of Spirulina to Craft Beer: Evaluation of the Effects on Volatile Flavor Profile and Cytoprotective Properties

**DOI:** 10.3390/antiox12051021

**Published:** 2023-04-28

**Authors:** Cosimo Taiti, Giovanni Stefano, Ester Percaccio, Silvia Di Giacomo, Matteo Iannone, Andrea Marianelli, Antonella Di Sotto, Stefania Garzoli

**Affiliations:** 1 Department of Agri-Food and Environmental Science, Università di Firenze, Sesto Fiorentino, 50019 Firenze, Italy; cosimo.taiti@unifi.it; 2 Department of Biology, Università di Firenze, Via Micheli 3, 50121 Firenze, Italy; giovanni.stefano@unifi.it; 3 Department of Physiology and Pharmacology “V. Erspamer”, Sapienza University of Rome, P.le Aldo Moro 5, 00185 Rome, Italy; ester.percaccio@uniroma1.it (E.P.); silvia.digiacomo@uniroma1.it (S.D.G.); antonella.disotto@uniroma1.it (A.D.S.); 4 Circolo ARCI La Staffetta, Via Don Minzoni 29, 56011 Calci, Italy; arcilastaffetta@gmail.com (M.I.); andreamarianelli93@gmail.com (A.M.); 5 Department of Chemistry and Technologies of Drug, Sapienza University, P.le Aldo Moro 5, 00185 Rome, Italy

**Keywords:** SPME-GC-MS, PTR-ToF-MS, chemical analyses, radical scavenger activity, oxidative stress, cytoprotective properties, Nrf2

## Abstract

SPME-GC-MS and PTR-ToF-MS techniques were applied to describe the content of volatile flavor compounds in a craft beer before and after adding spirulina. The obtained results showed that the volatile profile of the two beer samples differed. Furthermore, to chemically characterize biomass spirulina, a derivatization reaction followed by GC-MS analysis was performed, highlighting a high content of molecules belonging to different chemical classes, such as sugars, fatty acids and carboxylic acids. A spectrophotometric analysis of total polyphenols and tannins, investigation into the scavenging activity towards DPPH and ABTS radicals and confocal microscopy of brewer’s yeast cells were carried out. Moreover, the cytoprotective and antioxidant properties towards the oxidative damage induced by tert-butyl hydroperoxide (tBOOH) in human H69 cholangiocytes were investigated. Finally, the modulation of Nrf2 signaling under oxidative stress conditions was also evaluated. Both samples of beer were shown to contain similar levels of total polyphenols and tannins, with slightly increased levels in that containing spirulina 0.25% *w*/*v*. Moreover, the beers were found to be endowed with radical scavenging properties towards both DPPH and ABTS radicals, albeit with a weak contribution of spirulina; however, a higher riboflavin content was detected in spirulina-treated yeast cells. Conversely, the addition of spirulina (0.25% *w*/*v*) appeared to improve the cytoprotective properties of beer towards tBOOH-induced oxidative damage in H69 cells and reduce intracellular oxidative stress. Accordingly, the cytosolic Nrf2 expression was found to be increased.

## 1. Introduction

Beer is a complex mixture of components, produced from a variety of raw materials such as water, yeast, malt and hops, and contains a large number of chemical compounds, but only a small number of these compounds are “flavor-active”, i.e., directly involved in the production of flavor sensation when the product is consumed. The volatile fraction can be composed of countless compounds belonging to different chemical classes, including higher alcohols, esters, fatty acids, carbonyl compounds, sulfur compounds and furans [1]. 

The growing demand for a protein supply for human nutrition has led to the exploration of new and alternative protein sources such as unicellular proteins (SCPs). Many micro-organisms (algae, bacteria, yeast/filamentous fungi) can be used as a source of SCP, but due to their low nucleic acid content and high level of essential amino acids, algae are preferred over fungi and bacteria as a source of SCPs for human consumption, so the world production of microalgal biomass is steadily increasing [2,3].

The cyanobacterium *Arthrospira platensis* (known commercially as spirulina) is an edible, symbiotic, multicellular, filamentous blue-green microalgae that belongs to the cyanobacteria group (i.e., bacteria that fix nitrogen from the air) [4], and it is used as a food by Mexican and African people [5]. Currently, it is produced commercially in large open ponds under controlled conditions and is widely used as a safe functional food.

The potential health benefits of spirulina are mainly due to its chemical composition, which includes proteins, carbohydrates, essential amino acids, minerals (especially iron), essential fatty acids and vitamins [6,7]. Precisely thanks to its high protein content (it can vary from 50% to 70% [8]), spirulina is added to many foods such as isotonic drinks, cereal bars, instant soups, puddings, powdered preparations for cakes and biscuits and bread sticks [9]. Spirulina powder has been also used as an ingredient for chewable wafers to make chocolate bars and biscuits [10].

Most cyanobacteria, including spirulina, are capable of synthesizing vitamin B12 [11], also known as cobalamin, a water-soluble vitamin involved in the metabolism of every cellular function in the human body, and other vitamins such as vitamin K, vitamin A, tocopherol isomers (vitamin E) and metabolic intermediates [12].

Among microalgal pigments, phycobiliproteins (phycocyanin and phycoerythrin) play an important role in the commercial and biotechnological fields, with wide applications as natural dyes, dietary supplements and antioxidants, and in the biomedical and pharmaceutical fields as well [13]. The phycocyanobilin in spirulina can function as a NOX inhibitor. It is also a powerful free radical scavenger with the ability to scavenge hydroxyl, hydroxyl, peroxyl, hypochlorite and peroxynitrite radicals [14].

The use of spirulina protein concentrates has also been proposed as an alternative ingredient for the formulation of dietary supplements [15].

Additionally, in the cosmetic sector, a product based on a spirulina extract has been used for its skin properties, such as anti-aging, bringing benefits for the face and body [16]. It is also used as a pigment in lipsticks, eyeliners and eye shadows, and as an antioxidant and thickening agent in skin and hair care products and sunscreen [17].

In the United States, the FDA has classified spirulina extract as a certification-exempt color additive and approved its use in confectionery and other foods, while currently, in the European Union, spirulina extract is classified as a food colorant.

Over the years, beer has attracted great attention for its nutritional value and for its particular taste; in line with the growing interest in foods and beverages capable of combining nutritional value and healing properties, improvements in the composition of beer, through the addition (e.g., microalgae, plant extracts, fungi, probiotics, etc.) or removal (e.g., alcohol, gluten, or carbohydrates) of some compounds, have been obtained, thus leading to the production of “functional beverages” [18]. Despite the potential benefits of the biomass spirulina, however, variations in the sensory characteristics and acceptance of the finished product can arise. Hence, the interaction between spirulina and yeast are of utmost importance for understanding how best to use this new beneficial additive and how it interacts with volatile organic compounds (VOCs) during the fermentation process.

This research aimed to evaluate the effects of the addition of spirulina during the production phase of craft beer. In particular, in order to determine possible changes in the volatile chemical profile, two complementary analytical techniques, SPME-GC-MS and PTR-ToF-MS, were used. The content of polyphenols and tannins and the antioxidant power were measured, and confocal microscopy of brewer’s yeast cells was performed in order to detect the riboflavin content. In addition, the cytoprotective effect towards the oxidative damage induced by tert-butyl hydroperoxide (tBOOH) in human H69 cholangiocytes was studied.

To the best of our knowledge, this is the first work exploring the effects on volatile profile, flavor and cytoprotective properties of the addition of spirulina to a commonly used alcoholic beverage, highlighting a further beneficial effect of this microalga known for its high bioactive potential.

## 2. Materials and Methods

### 2.1. Production Process

The grist (ground malt grains) mixtures (American pale ale with fresh hop) were composed of 87% Pale Ale (Weyermann Brennerstraße 17-19D-96052 Bamberg) as base malt, 11% Cara Pils (Weyermann) for more body and foam and 2% acid malt for lower acidity of beer wort (Weyermann acidulated malt). The mashing process of the ground grains (grains:water in a 1:3 ratio) was performed in a multi-step system. Once the mixture had reached 45 °C, the temperature program proceeded as follows:

(1) 45 °C for 10 min (protease enzymes react to hydrolyze low-weight protein as nourishment for yeast);
(2) 66 °C for 40 min (β-amylase activity, pH 5.0–5.5, enzymatic synergy points between amylases);
(3) 72 °C for 20 min (α-amylase activity, pH 5.6–5.8, maximum activity);
(4) 78 °C for 5 min (enzymatical inactivation phase).

After 15 min of cooling, the filtering took place, with the washing of the threshes and the collection of the wort in a sanitized fermenter. This process was repeated 6 times, with water at pH 6. The wort boiling phase was performed for seventy minutes, together with the bitter and aroma hopping. 

Cascade hop pellets, due to there bitter attributes, were added at the start of the boiling phase (20 g in 37 L beer wort). During the last ten minutes of boiling, four different types of fresh hop inflorescences (Chinook, Centennial, Cascade, Willamette—30 g for each type), were also added.

The beer wort was then cooled using a plate heat exchanger, where the hot wort and the coolant (tap water) circulated in opposite directions. The must was then oxygenated to favor the start of fermentation by stirring for at least a couple of minutes. Finally, the yeast (Fermentis SafAle™ US-05, Lesaffre, Cedex, France) was inoculated and the mix was stirred again. The mix was closed in the fermenter for 12 days at 20 °C, with a gradual temperature decrement down to 4 °C. Afterwards, the bottling and priming processes were performed. The bottles were stored at 22–25 °C for 20 days, then the nucleation of carbon dioxide was repeated by placing the bottles in a refrigerator at 4 °C for 4–5 days. In this last phase, 0.8 gr/L of organic spirulina (about 0.4 g per single 50 mL bottle) was added.

### 2.2. Materials

Dried biomass of *Athrospira platensis* (thallus) was purchased from Livegreen Farm s.r.l., 09170 Oristano, Italy. Fresh hop inflorescences and hop pellets were purchased from Organic Farm Alpe di Puntato (Stazzema, LU, Italy) and Mr. Malt^®^ P.A.B. s.r.l. (Pasian di Prato, UD, Italy), respectively.

### 2.3. Chemicals

All the reagents used for the antioxidant activity assays, including Folin–Ciocâlteu phenol reagent, sodium carbonate (Na_2_CO_3_; 99% purity), tannic acid (Ph Eur purity), 1,1-diphenyl-2-picryl-hydrazyl (DPPH; 95% purity), 2,2′-azino-bis(3-thylbenzothiazoline-6-sulfonic acid) diammonium salt (ABTS; 98% purity), 2,2′-azobis (2-methylpropionamidine) dihydrochloride (AAPH; 97% purity), Trolox (97% purity), tert-butyl hydroperoxide solution (tBOOH; 80% purity) and the solvent ethanol (EtOH; 99.5% purity), were purchased from Merck (Darmstadt, Germany). To perform the assays, Trolox and DPPH were dissolved in EtOH, and ABTS and AAPH were prepared in phosphate-buffered saline (PBS; 0.1 M; pH = 7.0), sodium carbonate in sodium hydroxide (0.1 N) and tBOOH in water.

### 2.4. SPME Sampling

To describe the volatile chemical composition of beers, the sampling was carried out using the SPME technique. Small amounts of beer (~2 mL) were individually placed into a 20 mL glass vial with PTFE-coated silicone septum. For the extraction of volatiles, a SPME device from Supelco (Bellefonte, PA, USA) with 1 cm fiber coated with 50/30μm DVB/CAR/PDMS (divinylbenzene/carboxen/polydimethylsiloxane) was used. Before use, the fiber was conditioned at 270 °C for 30 min. Both beers were conditioned in a thermostatic bath at 60 °C for 30 min before sampling. Later, the fiber was exposed to the headspace of the samples for 20 min at 50 °C to collect the volatile compounds. Lastly, the SPME fiber was inserted into the injection port at 250 °C for the desorption of the extracted analytes.

### 2.5. GC-MS Analisys of Beers

The analyses were performed by using a gas chromatograph coupled with a mass spectrometer Clarus 500 model Perkin Elmer (Waltham, MA, USA), equipped with an FID (flame detector ionization). The used capillary column was a Varian Factor Four VF-1 [19,20]. The oven temperature was programmed as follows: initially 55 °C, then increased to 220 °C at 6°/min and finally held for 15 min. Helium was used as carrier gas at a constant rate of 1 mL/min. The mass spectrometer was operated at 70 eV (EI) in full scan mode in the range 40–450 *m*/*z*. The ion source and the connection parts’ temperature was 200 °C.

The identification of volatile compounds was performed by matching their mass spectra with those stored in the Wiley 2.2 and Nist 02 mass spectra library databases and by calculating the linear retention indices (LRIs) using a series of alkane standards analyzed under the same conditions as those of the samples. LRIs were then compared with available retention data reported in the literature. The peak areas of the FID signal were used to calculate the relative concentrations of the components expressed as a percentage without the use of an internal standard or any factor correction. All analyses were carried out in triplicate.

### 2.6. GC-MS Analysis of Spirulina after Derivatization

To describe the chemical composition of spirulina, a derivatization reaction was performed. For this purpose, ~2 mg of spirulina was added to 300 µL of pyridine and 100 µL of bis-(trimethylsilyl) trifluoroacetamide (BSTFA) (Sigma-Aldrich, Steinheim, Germany), with heating at 80 °C for 30 min. Then, 1 μL of the silylated sample was manually injected at 270 °C into the GC injector in splitless mode. The analysis was performed using the same apparatus GC-FID/GC-MS and the same capillary column (Varian Factor Four VF-1). The oven temperature program was as follows: 60 °C, then a gradient of 7 °C/minute to 170 °C for 1.0 min and a gradient of 8 °C/min to 250 °C for 25 min. Mass spectra were acquired in electron impact mode. The identification of compounds was based on the percentage of similarity plus comparison of mass spectra (MS) using the software NIST data library, with the percentage of total ion chromatograms (TIC%). Relative percentages for the quantification of the components were calculated through electronic integration of the GC-FID peak areas, and no response factors were calculated.

### 2.7. PTR-ToF-MS Analysis of Beers

To evaluate the beer headspace, commercial PTR-ToF-MS 8000 apparatus provided by Ionicon Analytik GmbH (Innsbruck, Austria)was used in its standard configuration (V mode). For each measurement, the drift tube ionization conditions were the following: 110 °C, 2.30 mbar, 550 V. This led to an E/N ratio of about 140 Townsend (1 Td = 10–17 V·cm^2^). The inlet line was heated at 110 °C and the inlet flow was set at 50 sccm. Before starting the measurements, all samples were brought to 30 °C. Mass spectra were recorded in the mass range between 20 and 220, with one mass spectrum acquired every second. Data were recorded using TOFDAQ v.183 (data acquisition software, Tofwerk AG, Thun, Switzerland) and are expressed as ppbv (parts per billion by volume). Thus, 10 mL of beer (control beer and spirulina beer, respectively) was placed in a 0.5 L glass jar and flushed for 60 s with cleaned air (zero air generator), then each sample was analyzed for 100 s. All samples were replicated tree times. As reported by Aprea et al. [21], when samples are characterized by high ethanol emission, inert gas dilution needs to be applied (with an inert gas to sample ratio of 2:1) before starting the measuring with the aim of preventing primary ion depletion and the formation of ethanol clusters, which might affect the final quantification of volatiles. However, since the parent ethanol peak (*m*/*z* 47.049) was saturated during our analysis, the ethanol emission monitoring was assessed following the values of the main ethanol-related peaks (*m*/*z* 65.059 and 75.080) as previously suggested by Capozzi et al. [22]. Subsequently, only peaks whose average signal was higher than 1 ppbv were reported, while the peaks related to ethanol and ethanol clusters were discarded (*m*/*z* 29, 30, 32, 34, 37, 39, 46, 47, 48, 55, 66, 76, 93, 94,121, 122, 139), as reported by Boscaini et al. [23].

### 2.8. Spectrophotometric Analysis of Total Polyphenols and Tannins 

The total polyphenols and tannins were determined by using the Folin–Ciocâlteu method, as previously reported [24]. Specifically, the tannin amount was determined by using the difference between the total polyphenols in beers and in their supernatants after precipitation with polyvinylpyrrolidone (PVPP; 100 mg per ml of beer). Briefly, 20 μL of tested samples, 100 μL of the Folin–Ciocâlteu reagent and 80 μL of a sodium carbonate solution (7.5% *w*/*v*) were mixed and incubated for 2 h, then the absorbance was read at 765 nm using an Epoch Microplate Spectrophotometer (BioTek, AHSI, Milan, Italy). The total content of polyphenols and tannins was calculated as tannic acid equivalents (TAE) per mg of beer [25].

### 2.9. Radical Scavenging Activity

The DPPH and ABTS radical scavenger power of the tested samples was evaluated using previously published methods with minor changes [26]. With regard to the DPPH assay, progressive dilutions of the tested samples (20 µL) were added to a 0.1 mM DPPH radical solution (180 µL), then the plate was incubated in the dark at room temperature for 30 min. ABTS was activated to its radical cation by mixing equal volumes of 5 mM ABTS and 2 mM AAPH solutions in phosphate-buffered saline (PBS) at 68 °C for 45 min. Thereafter, the radical cation (180 µL) was mixed with progressive dilutions of the tested samples (20 µL) and incubated at 37 °C in the dark for 10 min. The absorbance of DPPH and ABTS radicals was measured at 517 nm and 734 nm, respectively, using an Epoch Microplate Spectrophotometer (BioTek, AHSI, Milan, Italy). In each experiment, suitable controls were added to evaluate the maximum absorbance of the radicals and the presence of possible interfering compounds in the tested samples; Trolox was used as positive control. The radical scavenger activity was calculated as a percentage of the control; the IC_50_ (half maximal inhibitory concentration) value was estimated from the concentration–response curves, using the Hill equation [25].

### 2.10. Confocal Microscopy of Beer Yeast Cells

A confocal microscope was used to obtain fluorescence spectra relative to autofluorescence relative to riboflavin [27]. Spectral images were recorded using a Leica TCS SP5 confocal microscope (Leica Microsystems CMS, Wetzlar, Germany) equipped with an acusto-optical beam splitter and an upright microscope stand (DMI6000). A drop of beer or beer with spirulina was imaged using a 40× objective (HCX PL APO OIL UV) and images with an area of 98 × 98 mm were captured. The pinhole was set to one ‘Airy unit’. Fluorescence spectra of riboflavin were recorded (over the 510–770 nm waveband, using the Leica LAS-AF software package) through measurements in λ-scan mode with a detection window of 10 nm and a 488 nm laser as the excitation line. The area of yeast cells was measured using Fiji (https://imagej.net/Fiji accessed on 28 March 2023). Values are presented as mean SEM of the number of the indicated determinations. Statistical significance was determined using an unpaired *t*-test (PRISM 9.0; GraphPad Software), with *p* ≤ 0.005 considered significant.

### 2.11. Cytoprotective Activity towards Oxidative Stress

#### 2.11.1. Cell Culture

Human H69 intrahepatic cholangiocytes, a SV40-immortalized human nonmalignant bile duct epithelial cell line, originally derived from a normal liver harvested for transplantation [28], were kindly gifted by Prof. Romina Mancinelli (Department of Anatomical, Histological, Forensic and Orthopedic Sciences, Sapienza University of Rome, Italy). The cells were used as a model of the gastro-intestinal tract to study the possible cytoprotective effects of the tested samples when taken orally. They were cultivated at a density of about 2 × 10^4^ cells/cm^2^ under standard conditions (37 °C and 5% CO_2_), as previously reported [29], and treated when the logarithmic growth phase was reached.

#### 2.11.2. Cytotoxicity

The effect of the treatments on cell viability was determined after 24 h exposure through a 3-[4,5-dimethylthiazol-2-yl]-2,5-diphenyl tetrazolium bromide (MTT) assay, as previously reported [25]. Briefly, confluent cells were grown in 96-well microplates (2 × 10^4^ cells/well) for 24 h, then treated with increasing volumes of the beer samples, up to a maximum 1% *v*/*v* in the medium, for a further 24 h. Accordingly, spirulina and the positive control doxorubicin were assayed. Considering that the tested beers were 5% *v*/*v* alcohol grade, a maximum 0.05% (*v*/*v*) EtOH in cell medium was used as a vehicle control. The results are expressed as a percentage of the vehicle control. A reduction in cell viability higher than 30% with respect to the control was considered as a biologically significant cytotoxic effect of the treatments [30]. 

#### 2.11.3. Cytoprotection towards the Oxidative Damage induced by Tert-Butyl Hydroperoxide (t-BOOH)

The cytoprotective effects of the tested samples were evaluated with respect to the oxidative damage induced by a subtoxic concentration of tBOOH (500 μM), selected in previous experiments from the concentration–response curve, which induced about a 40% inhibition of cell viability. To perform the assay, 2 × 10^4^ cells/well were grown in a 96-well microplate for 24 h, then treated with the tested samples for 24 h and with the tested samples in the presence of tBOOH for a further 3 h. The effect of the treatments on the cell viability and intracellular levels of reactive oxygen species (ROS) was determined through MTT and 2,7-dichlorofluorescein diacetate (DCFH-DA) assays, respectively, as previously reported [25].

#### 2.11.4. Immunofluorescence Analysis of Nrf2

To perform the analysis, 2 × 10^4^ cells were seeded in a 24-well plate and treated with beer, beer with spirulina 0.25% *w*/*v* or spirulina at the corresponding concentration in the beer for 30 min, both in the absence and presence of tBOOH. After incubation, they were fixed in methanol and washed in phosphate-buffered saline + Tween 20 (PBS-T), incubated in 4% bovine serum albumin (BSA) and PBS-T, then stained using a NF-E2 (#ABE413, Merck Millipore, Germany) primary antibody and Hoechst (1 μg/mL) dye for 1 h at room temperature (RT). After washing in PBS-T, a suitable secondary antibody (Alexa Fluor 594-conjugated Chrom Pure Rabbit igG, Jackson Immuno Research Europe Ltd., Ely, UK) was added for 1 h in a dark room at RT. After washing, the cells were analyzed using a Cytation 1 Cell Imaging Multimode Reader (BioTek, AHSI, Milan, Italy) and the fluorescence quantified.

### 2.12. Statistical Analysis

Data are expressed as mean ± standard error (SE) of at least three experiments, in which each treatment was performed at least in triplicate. GraphPad Prism™ (Version 6.00) software (GraphPad Software, Inc., San Diego, CA, USA) was used to perform the statistical analysis and data representation. A statistically significant difference (*p* value < 0.05) between multiple treatments was evaluated by using one-way analysis of variance (one-way ANOVA), followed by Dunnett’s multiple comparison post test. The IC_50_ value was obtained from concentration–response curves, calculated by using the Hill equation [25]. 

## 3. Results

### 3.1. Vapor-Phase Beer Chemical Composition

The volatile profile of beers was described by SPME technique application. Thirteen compounds were found and identified (Table 1), among which the fatty acids were the more abundant fraction. Ethyl caprylate and ethyl caprate were the major compounds in both beers, even though all the identified esters reached higher mean percentage values in the beer without spirulina.

The only qualitative differences found between the two samples concerned the following compounds: propanal, 2-methyl,-oxime and 1H-1,2,4 -triazole were only characteristic of beer with spirulina. 

### 3.2. Chemical Composition of Spirulina after Derivatization

To obtain the chemical profile of spirulina, a direct injection analysis of the silylated biomass was performed. The results allowed the identification of twenty compounds belonging to different chemical classes, including carboxylic acids, fatty acids and sugars (Table 2). Lactic acid (64.7%) was the most abundant component. Six different sugars were identified, and five of them had comparable percentage values (from 1.5 to 2.3%). Glyceric acid (0.6%) and glycerol (2.9%) were also detected. Palmitic and stearic acid were the only two fatty acids found in the matrix. It is also of interest to note the presence of pulchelloside I, an iridoid glucoside, and of the terpene phytol, both being detected in spirulina biomass.

### 3.3. PTR-ToF-MS: Determination of Volatile Compounds from Beers

A general overview on the volatile composition was obtained from the PTR-ToF-MS analyses. In detail, 47 peaks from beer and 43 from beer with spirulina emerged, with different relative emission intensities (Table 3). Among these peaks, the *m*/*z* 113, *m*/*z* 165, *m*/*z* 177 and *m*/*z* 203 were detected only in beer without spirulina, while the others detected generally showed a reduced emission in beer with spirulina, highlighting how adding spirulina affects the final volatile profile. In contrast, the compound detected at *m*/*z* 147 showed a higher emission in beer with spirulina with respect to the other sample. 

**Figure 1:**
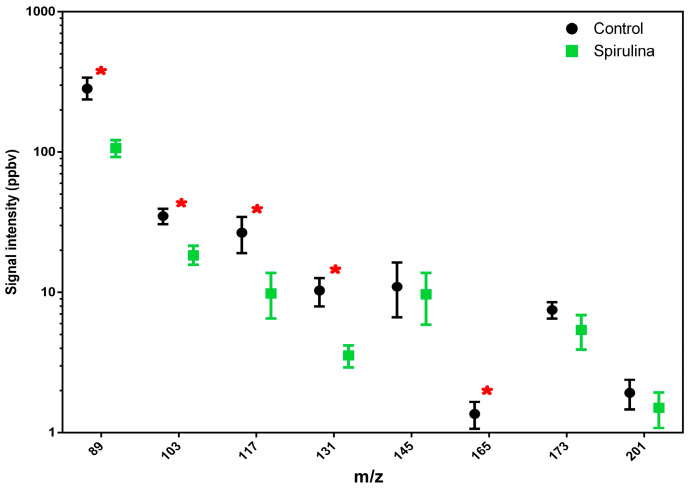
Total amount of esters released by different beer samples (beer vs. beer with spirulina) are reported. Values in the graphs are the mean of three replicates ± standard deviation. One-way ANOVA (Tukey’s test, *p* < 0.05 level) was used for the statistical analysis. Red asterisks highlight statistical differences.

Regarding the ethanol emission detected at *m*/*z* 65 and *m*/*z* 75, this was lower in beer with spirulina, as was the emission of the most significant flavor-active ester compounds in beer (Figure 1). In particular, the compounds detected at *m*/*z* 103, 117, 131 and 165 showed a significant difference between the two beer samples, with higher average values in beer without spirulina. 

### 3.4. Spectrophotometric Analysis of Total Polyphenols and Tannins

**Figure 2:**
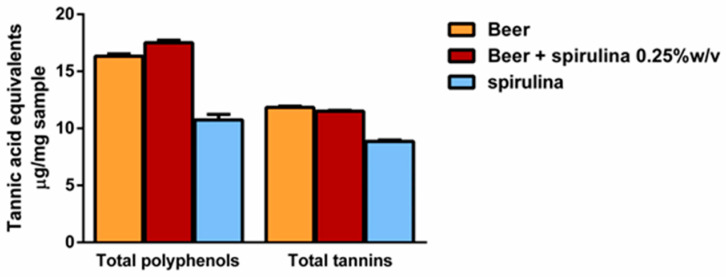
Levels of total polyphenols and tannins per mg of beer, beer with spirulina 0.25% *w*/*v* and spirulina. Data are expressed as the average ± standard error of at least two experiments with three replicates (*n* = 6).

Preliminarily, we established that 1 mL of beer weighed 984.2 mg and 986.7 mg after adding spirulina 0.25% *w*/*v*. The levels of total polyphenols and tannins were determined per mg of sample, which corresponded to about 1 µL of the sample. The results showed the presence of similar levels of both polyphenols and tannins in the beers, with a slight but not statistically significant polyphenol increase in the sample with spirulina 0.25% (Figure 2). Similarly, 1 mg spirulina contained about 60% and 75% of the level of polyphenols and tannins of the beer. However, considering that it was 0.25% *w*/*v* in the beer, it is expected that it only slightly affected the total polyphenol and tannin content.

### 3.5. Radical Scavenging Activity

Under our experimental conditions, both samples of beer were found to be endowed with radical scavenging properties, although with a higher potency towards ABTS (Figure S1 and Table 4); moreover, the sample of beer with spirulina 0.25% *w*/*v* was almost three times more potent than the beer alone, as also confirmed by the IC_50_ values (Table 4). Under the same experimental conditions, the corresponding concentrations of spirulina in the beer exhibited a slight DPPH scavenger activity, achieving a maximum of 40% inhibition at the highest concentration tested (0.25% *w*/*v* of 100 µL/mL, corresponding to 250 µg/mL); conversely, the extract was ineffective against ABTS (Figure S1).

As expected, the positive control Trolox showed the most potent scavenging activity in all the assays; indeed, considering that the IC_50_ values of the beer samples were about 50 and 5 µL/mL (corresponding to about 50 and 5 µg/mL) in the DPPH and ABTS assays, respectively, the potency of Trolox as a scavenging agent was at least 1000-fold higher than that of the beers (Table 4).

### 3.6. Confocal Microscopy Analysis

**Figure 3:**
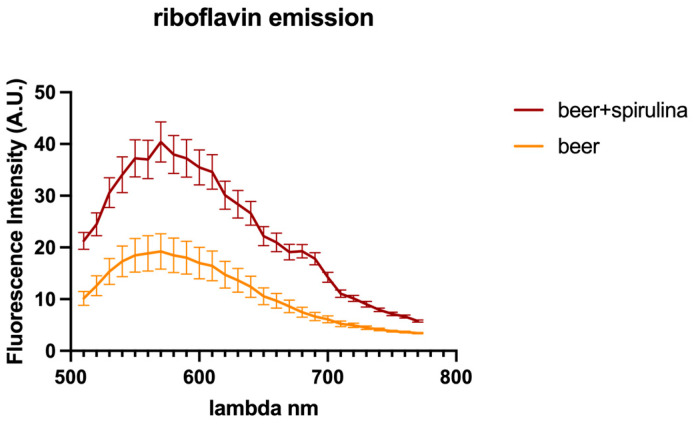
Riboflavin autofluorescence emission spectra detected in yeast cells.

**Figure 4:**
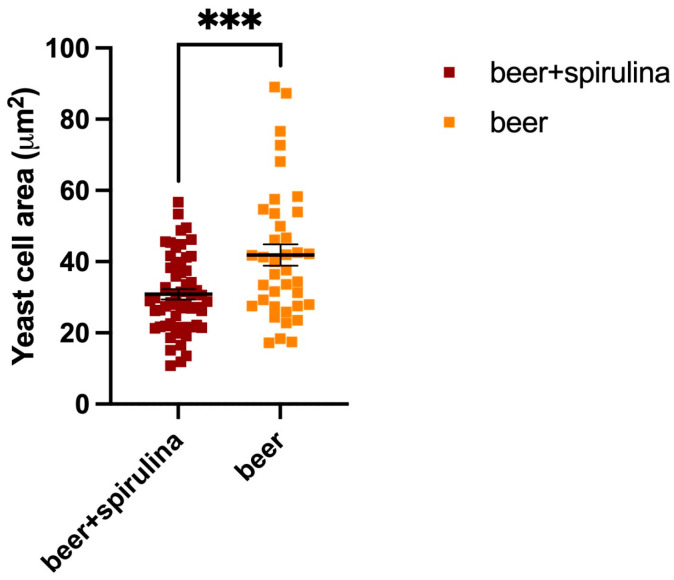
Yeast cell areas in both the sample beer and the beer that was augmented with spirulina; ***: Statistical analysis was performed using Student’s *t*-test (*** *p* ≤ 0.001).

In this paper, we explored the confocal autofluorescence of riboflavin in yeast cells in the absence and presence of spirulina. The amount of riboflavin identified in the beer with spirulina is nearly double that found in the sample without spirulina (Figure 3). The impact of such an increase on yeast cells was also investigated, and the findings highlighted that yeast cells co-cultivated with spirulina had a smaller total cell area than cells not co-cultivated with spirulina (Figure 4). 

### 3.7. Antioxidant Cytoprotective Activity

**Figure 5:**
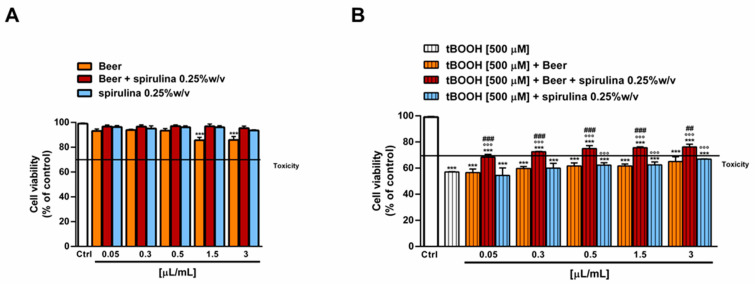
Effect of beer, beer with spirulina 0.25% *w*/*v* and spirulina 0.25% *w*/*v* at the corresponding concentrations in the beer on the H69 cell viability in the absence (**A**) and presence (**B**) of the prooxidant agent tBOOH. Data represent the average and standard error of at least three independent experiments (*n* = 3). *** *p* < 0.001, significant difference with respect to control (ANOVA followed by Dunnett’s multiple comparison post test). °°° *p* < 0.01, significant difference with respect to tBOOH (ANOVA followed by Dunnett’s multiple comparison post test). ### *p* < 0.001, significant difference of beer + spirulina 0.25% *w*/*v* with respect to beer (Student’s *t*-test).

**Figure 6:**
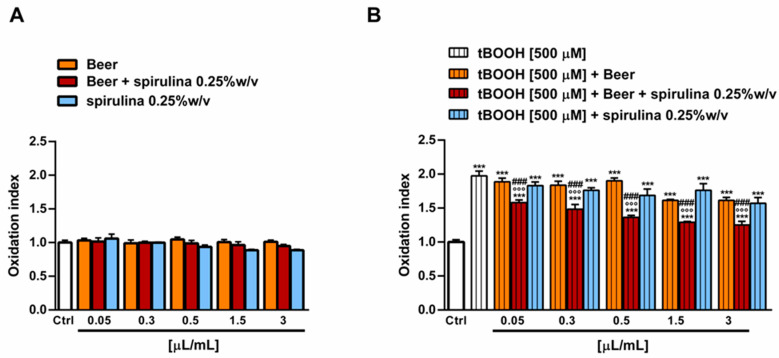
Effect of beer, beer with spirulina 0.25% *w/v* and spirulina 0.25% *w/v* at the corresponding concentrations in the beer on the levels of reactive oxygen species (ROS) in the absence (**A**) and presence (**B**) of the prooxidant agent tBOOH in H69 cells. Data represent the average and standard error of at least three independent experiments (*n* = 3). *** *p* < 0.001, significant difference with respect to control (ANOVA followed by Dunnett’s multiple comparison post test). °°° *p*< 0.01, significant difference with respect to tBOOH (ANOVA followed by Dunnett’s multiple comparison post test). ### *p* < 0.001, significant difference of beer with spirulina 0.25% *w*/*v* with respect to beer (Student’s *t*-test).

The antioxidant power of beer, beer with spirulina 0.25% *w*/*v* and spirulina at the corresponding concentrations in the beer (0.25% *w*/*v*) was also evaluated in a biological system with the pro-oxidant damage induced by tBOOH. Specifically, the H69 cells were exposed to a 24 h pre-treatment with the beer samples, followed by a 3 h co-treatment in the presence of tBOOH. Under our experimental conditions, the beer samples were nontoxic up to the concentration of 3 µL/mL (Figure 5A) and induced no signs of oxidative stress (Figure 6A), while cytotoxic effects were registered at higher concentrations (data not shown). 

Therefore, the cytoprotective properties of the tested samples towards tBOOH were evaluated up to 3 µL/mL. As displayed in Figure 6B, tBOOH significantly affected the cell viability, lowering it by almost 45% with respect to the control; accordingly, the ROS levels were doubled by the treatment (Figure 6B). The sample of beer did not counteract the tBOOH damage, despite significant cytoprotective effects of the beer with spirulina 0.25% *w*/*v*, which achieved a maximum of a 20% increase in the cell viability with respect to tBOOH at the highest tested concentrations. Similarly, the intracellular ROS levels were reduced by up to 1.5 times with respect to tBOOH, although without achieving the basal levels. A slight increase in cell viability, albeit without a significant ROS reduction, was also induced by the highest concentrations of spirulina, thus suggesting a possible contribution of the extract to the beer’s antioxidant activity (Figure 5B and Figure 6B).

**Figure 7:**
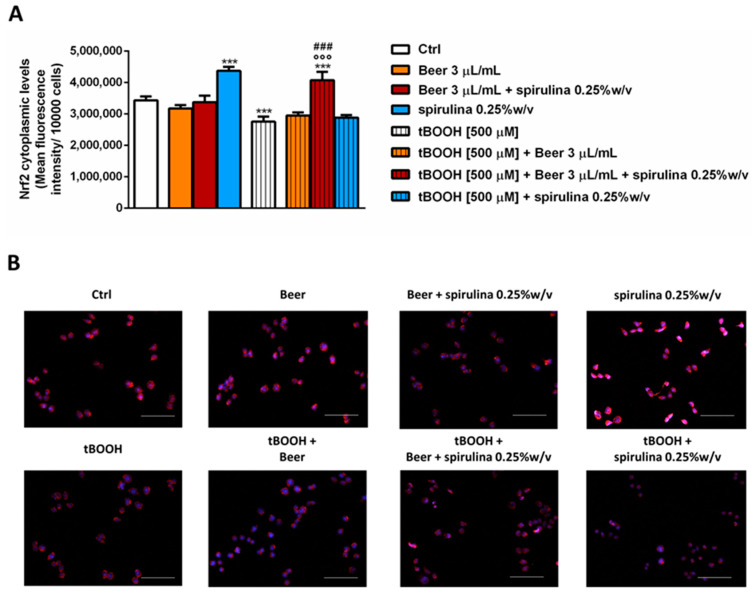
Effects of beer, beer with spirulina 0.25% *w*/*v* and spirulina at the corresponding concentrations in the beer on the Nrf2 cytoplasmic expression both in the absence and presence of tBOOH in H69 cells. (**A**): Bar graph of the Nrf2-fluorescence. Data displayed as mean ± SE of at least three independent experiments with at least two technical replicates (*n* = 9). (**B**): Representative images of cells stained with suitable antibodies and Hoechst 33258 dye. *** *p* < 0.001, significant difference with respect to control (ANOVA followed by Dunnett’s multiple comparison post test). °°° *p* < 0.01, significant difference with respect to tBOOH (ANOVA followed by Dunnett’s multiple comparison post test). ### *p* < 0.001, significant difference of beer + spirulina 0.25% *w*/*v* with respect to beer. Scale bars = 20 µm.

Finally, we explored the ability of the tested samples to modulate the cytoplasmic expression of Nrf2, a known intracellular factor activated during stress conditions in order to activate the cellular defense responses [31]. After 30 min exposure, the samples of beer (3 µL/mL) did not affect the basal levels of Nrf2, despite a significant increase induced by the corresponding concentration of spirulina (7.5 µg/mL); conversely, tBOOH induced a significantly reduced expression of Nrf2, which was restored and also increased (up to 20% with respect to tBOOH) by beer with 0.25% *w*/*v* spirulina compared to the control (Figure 7A,B). Surprisingly, the beer and spirulina alone were unable to counteract the damage induced by tBOOH, with the Nrf2 expression being comparable to that of tBOOH.

The obtained data suggest that adding spirulina to beer may confer cytoprotective properties to the sample, thus allowing the oxidative stress induced by tBOOH to be partly counteracted, likely through promoting the Nrf2 expression. 

## 4. Discussion

Many compounds contribute to the flavor and aroma of beer, and sometimes just a little difference in their intensity emission may produce an entirely different flavor. 

In this paper, the volatile chemical composition of beer before and after spirulina addition was investigated through SPME-GC/MS and PTR-ToF-MS. Our results showed that the volatile profiles of the two beer samples differed for a series of compounds.

With the aim to better compare the volatile compounds emitted in the presence of spirulina, we focused our attention on the volatile organic compounds (VOCs) with the most aromatic impact on beer. Therefore, all signals linked to terpene emission were discarded from the discussion because they are linked to the addition of hops [32].

The obtained results with both survey techniques used showed that some signals and their intensity emission were different between beer with and without spirulina. For example, the reduced ethanol emission in beer with spirulina highlighted a lower fermentative performance of the yeast when adding spirulina during the fermentative process. This finding is likely linked with the antimicrobic spirulina effect that may have interfered with the fermentation process reported by some authors [33,34].

Similarly, since ester compounds synthesized through the complex metabolic pathways of yeasts play an important role in the final flavor of beers, we tried to gain a clear understanding of the interaction between ester production and spirulina addition [35]. In particular, compounds such as ethyl acetate (*m*/*z* 89; solvent-like aroma), isoamyl acetate (*m*/*z* 131; banana aroma), ethyl caproate (*m*/*z* 145; aniseed, apple-like aroma), phenyl ethyl acetate (*m*/*z* 165; roses, honey), ethyl caprylate (*m*/*z* 173; sour apple aroma) and ethyl caprate (*m*/*z* 201; floral odor) showed an higher emission in the sample without spirulina. Furthermore, the emission peaks linked to ester compounds clearly indicated a different behavior for the two analyzed beers, highlighting how the spirulina addition reduced the fermentative activity of yeasts. 

Our chemical analyses conducted on spirulina biomass showed the presence of a high amount of lactic acid. Generally, the addition of lactic acid after the primary fermentation makes the beer more acidic due to the effect of the *Lactobacillus* bacteria. In fact, *Lactobacillus* is part of a family of bacteria called “lactic acid bacteria”. The addition of spirulina, in a limited percentage, during the fermentation process allows the creation of a tasty beer but with undoubted alterations in smell and taste compared to conventional beer [34]. 

From a functional point of view, in the present study, we evaluated the beneficial properties derived from the addition of spirulina to beer. In general, its consumption has been associated with antioxidant, anti-inflammatory, hypoglycemic, lipid-lowering and immunomodulatory effects [36,37,38,39]. Recently, it has also been reported to inhibit the oxidative response induced by highly active antiretroviral therapy in HepG2 cells, likely through the modulation of Nrf2 signaling [4]. In line with this evidence, we evaluated the effects of adding spirulina to craft beer in terms of antioxidant power and improvements in cellular defenses in a model of human cholangiocytes.

The obtained results showed that the spirulina extract contained polyphenolic compounds, including a high amount of tannins. These data are in agreement with the results obtained for both *Arthrospira platensis* and *Spirulina maxima* [40,41,42]. However, spirulina had little effect on total polyphenol and tannin levels in both beer samples.

Beer is generally characterized by the presence of polyphenolic compounds, such as phenolic acids and tannins, flavones and flavonols [43], which contribute to its typical organoleptic characteristics and stability as well as to its antioxidant properties [44,45,46]. We confirmed similar polyphenol levels and radical scavenging properties in our beer samples; a greater scavenging capacity against the ABTS radical was also highlighted for beer with 0.25% *w*/*v* spirulina compared to beer, thus suggesting that some compounds found in spirulina, such as fatty acids and/or sugars, may contribute to the increase in antioxidant power [47,48].

Using confocal microscopy, riboflavin autofluorescence can be observed in yeast cells, providing relevant information on this important cofactor [27]. In general, riboflavin fluorescence is influenced by several factors, including pH and the presence of other molecules [49]. Through this technique, it is possible to trace the amount of riboflavin inside the yeast cells under different conditions.

Because riboflavin is known to play a vital role in ROS detoxification through its ability to act as an antioxidant, the obtained data in our investigation suggest that cells treated with spirulina are presumably more stressed than yeast cells grown without spirulina. Therefore, it can be inferred that beer with spirulina contains more riboflavin, which may be beneficial.

The sample of beer with spirulina 0.25% *w*/*v* also showed increased cytoprotective properties towards the oxidative damage induced by tBOOH, improving the cell viability and lowering the intracellular ROS levels. To the best of our knowledge, this is the first study exploring the cytoprotective antioxidant properties of beer before and after the addition of spirulina. A previous study showed the ability of an alcoholic dark beer extract to confer protection against the oxidative damage of hydrogen peroxide in brain cell models [50].

Our results also showed that spirulina was able to increase the basal cytosolic Nrf2 levels, although without counteracting the lowering induced by tBOOH; moreover, it strengthened the ability of beer to counteract the oxidative damage of tBOOH and to increase the cytosolic levels of Nrf2. Our results are in agreement with previous studies that highlighted the ability of spirulina to upregulate the expression of Nrf2 and detoxification antioxidant genes, such as CAT and NQO-1 [4,37,51]. Although future studies are required to clarify the kind of interaction between spirulina and beer and the mechanisms of action, adding spirulina seems to be an interesting strategy to protect hepatic cells from oxidative damage via an Nrf2 response stimulation.

## 5. Conclusions

In this work, the effects of the addition of spirulina to craft beer on the volatile profile and on the antioxidant and cytoprotective power, were evaluated. Chemical analyses showed that the composition of beer was altered with the addition of spirulina in terms of the content of compound esters known to be partly responsible for the aroma and flavor of the beer. Biological assays demonstrated that even though the antioxidant power was slightly increased in beer with spirulina, greater effects were observed on the cytoprotective properties towards the oxidative damage. This work reported valuable information regarding the potential beneficial effects of microalgae, and based on the obtained results, it could be important to further explore its effects on food products.

## Figures and Tables

**Table 1:** Chemical composition (percentage mean values ± standard deviation) of vapor-phase beer samples.

N°	COMPONENT ^1^	LRI ^2^	LRI ^3^	Bc (%)	Bs (%)
1	ethyl acetate	601	599	5.0 ± 0.02	0.5 ± 0.02
2	1-butanol, 3-methyl	744	740	11.2 ± 0.03	8.9 ± 0.03
3	1-butanol, 2-methyl	748	744	8.4 ± 0.02	8.0 ± 0.02
4	propanal, 2-methyl,-oxime	795	797	-	10.5 ± 0.04
5	isoamyl acetate	861	859	2.8 ± 0.02	0.5 ± 0.01
6	1H-1,2,4-triazole	877	869	-	2.4 ± 0.02
7	2-methyl butyl acetate	882	879	0.8 ± 0.01	-
8	ethyl caproate	998	996	2.4 ± 0.02	2.2 ± 0.02
9	ethyl caprylate	1183	1181	33.5 ± 0.06	32.9 ± 0.06
10	phenyl ethyl acetate	1233	1229	2.3 ± 0.02	1.6 ± 0.02
11	ethyl caprate	1385	1382	28.5 ± 0.05	26.9 ± 0.05
12	phenyl ethyl butyrate	1445	1440	0.8 ± 0.01	0.5 ± 0.01
13	ethyl laurate	1590	1581	4.3 ± 0.02	3.4 ± 0.02
	SUM			100.0	98.3

^1^ The components are reported according to their elution order on apolar column; ^2^ linear retention indices measured on apolar column; ^3^ Linear retention indices from the literature. Bc: percentage mean values of beer components (%); Bs: percentage mean values of beer with spirulina components; -: not detected.

**Table 2:** Chemical composition (percentage values) of spirulina after derivatization.

N°	COMPONENT	Spirulina (%)
**CARBOXYLIC ACIDS**		
1	lactic acid	64.7
2	malonic acid	0.3
3	succinic acid	4.2
4	L-idopyranuronic acid	0.2
**FATTY ACIDS**		
5	palmitic acid	2.1
6	stearic acid	0.5
**SUGARS**		
7	L-rahmnose	2.2
8	D-xylose	2.3
9	arabinofuranose	2.3
10	D-lyxofuranose	1.5
11	3-α-mannobiose	1.8
12	D-galactofuranoside	0.2
**SUGAR ACIDS**		
13	glyceric acid	0.6
**SUGAR ALCOHOLS**		
14	glycerol	2.9
**TERPENES**		
15	phytol	0.4
**OTHERS**		
16	2,3-butanediol	0.2
17	hexadecane	8.0
18	2-pentanol	0.3
19	cyclohexanol, trans-	0.8
20	pulchelloside I	0.9

Percentage values of the components of spirulina biomass after derivatization.

**Table 3:** Compounds identified via PTR-ToF-MS: mass/charge (*m*/*z*) ratios, chemical formula, emission average and SD for each VOC from different beer samples. At the bottom, the total VOC emission and the total number of signals detected are reported.

*M/Z*	Chemical Formula	Beer		Beer with Spirulina	
		Average	SD	Average	SD
27	C_2_H_3_^+^	494.70	93.99	227.13	53.45
31	CH_3_O^+^	5.97	1.63	3.60	0.54
41	C_3_H_5_^+^	96.18	14.99	78.06	13.04
43	C_2_H_3_O^+^	1074.06	134.61	693.08	120.14
43	C_3_H_7_^+^	129.27	27.51	74.84	19.09
45	C_2_H_5_O^+^	44.68	4.04	20.88	6.12
57	C_4_H_9_^+^	120.94	18.81	151.64	20.31
59	C_3_H_7_O^+^	25.28	7.09	27.67	7.44
61	C_2_H_5_O_2_^+^	31.97	7.52	46.82	11.84
63	C_2_H_7_O_2_^+^	12.57	1.01	18.96	3.21
65	C_5_H_5_^+^	17.29	2.63	5.56	2.88
67	C_5_H_7_^+^	5.80	1.51	1.87	0.29
69	C_5_H_9_^+^	6.04	1.32	3.80	3.15
71	C_5_H_11_^+^	61.32	3.91	70.79	13.10
73	C_4_H_9_O^+^	13.05	1.88	10.57	3.77
75	C_4_H_11_O^+^	105.89	14.09	54.74	6.61
77	C_6_H_5_^+^	4.12	2.39	1.69	0.34
79	C_6_H_7_^+^	2.33	0.79	1.95	0.32
85	C_6_H_9_O^+^	5.91	0.85	2.27	0.93
87	C_5_H_11_O^+^	14.61	6.60	9.50	1.47
89	C_4_H_9_O_2_^+^	316.65	18.36	127.06	10.21
95	C_7_H_11_^+^	22.79	3.20	7.58	1.76
101	C_6_H_13_O^+^	28.14	6.68	15.93	3.21
103	C_5_H_11_O_2_^+^	38.78	6.36	18.06	5.68
105	C_8_H_9_^+^	12.17	1.19	8.60	3.07
107	C_8_H_11_^+^	16.50	3.79	6.37	1.13
109	C_8_H_13_^+^	3.68	0.93	1.83	0.38
113	C_6_H_9_O_2_^+^	4.63	1.18	0.00	0.00
115	C_6_H_11_O_2_^+^	8.14	2.62	1.90	0.71
117	C_6_H_13_O_2_^+^	27.57	8.22	9.47	3.00
131	C_7_H_15_O_2_^+^	10.15	3.32	3.74	1.02
135	C_10_H_15_^+^	10.11	0.83	5.44	0.44
145	C_8_H_17_O_2_^+^	12.38	4.19	10.17	4.11
147	C_9_H_7_O_2_^+^	3.89	0.85	8.13	0.73
161	C_10_H_9_O_2_^+^	5.82	1.12	1.92	0.25
173	C_10_H_21_O_2_^+^	5.51	1.00	3.51	1.52
177	C_9_H_21_O_3_^+^	8.34	2.44	0.00	0.00
189	C_8_H_13_O_5_^+^	14.13	2.31	2.77	0.35
201	C_12_H_25_O_2_^+^	1.60	0.38	1.39	0.20
203	C_12_H_11_O_3_^+^	3.44	1.14	0.00	0.00
**Total VOC emission**		2636.81		1928.87	
**Total number of signals**		40		37	

**Table 4:** IC_50_ values (µL/mL) of beer and beer + spirulina 0.25% *w*/*v*, and of spirulina 0.25% *w*/*v* in the beer and the positive control Trolox (µg/mL) in DPPH and ABTS radical scavenging activity assays.

Sample		DPPH Scavenging Activity	ABTS Scavenging Activity
Beer	IC_50_ (CL) µL/mL	57.7 (43.1–77.3)	5.4 (1.8–9.3)
Beer + spirulina 0.25% *w/v*		49.9 (25.2–95.0)	1.9 (0.7–4.9) ^#^
Spirulina	IC_50_ (CL) µg/mL	ne	ne
**Trolox**		7.0 (5.0–9.7)	5.8 (4.2–8.4)

CL: confidence limit; ne: not evaluable, with the achieved effect being lower than 80%; ^#^ *p* < 0.05, significant difference of beer + spirulina 0.25% *w*/*v* respect to beer (Student’s *t*-test).

## Data Availability

All data reported in this study are available within the article.

## Supplementary Materials

The following supporting information can be downloaded at: https://www.mdpi.com/article/10.3390/antiox12051021/s1, Figure S1: Radical scavenger activity of beer, beer + spirulina 0.25% *w*/*v* and spirulina at the corresponding concentrations in the beer (A,B) and of Trolox (C,D) towards DPPH and ABTS radicals.
