# Supplementary material for: Addition of Spirulina to Craft Beer: Evaluation of the Effects on Volatile Flavor Profile and Cytoprotective Properties
Source: Antioxidants (Basel). 2023 Apr 28;12(5):1021. doi: 10.3390/antiox12051021 (PMC10215288; doi:10.3390/antiox12051021)
Supplement: Supplementary file 1 [file antioxidants-12-01021-s001.zip › antioxidants-2354782-supplementary.pdf]

antioxidants

Supplementary materials

Addition of Spirulina to Craft Beer: Evaluation of the Effects on Volatile Flavor Profile and Cytoprotective Properties

Cosimo Taiti [1], Giovanni Stefano [2], Ester Percaccio [3], Silvia Di Giacomo [3], Matteo Iannone [4], Andrea Marianelli [4], Antonella Di Sotto [3,†] and Stefania Garzoli [5,*,†]

[1] Department of Agri-Food and Environmental Science, Università di Firenze, Sesto Fiorentino, 50019 Firenze, Italy; cosimo.taiti@unifi.it
[2] Department of Biology, Università di Firenze, via Micheli 3, 50121 Firenze, Italy; giovanni.stefano@unifi.it
[3] Department of Physiology and Pharmacology "V. Erspamer", Sapienza University of Rome, P.le Aldo Moro 5, 00185 Rome, Italy; ester.percaccio@uniroma1.it (E.P.); silvia.digiacomo@uniroma1.it (S.D.G.); antonella.disotto@uniroma1.it (A.D.S.)
[4] Circolo ARCI La Staffetta, Via Don Minzoni 29, 56011 Calci, Italy; arcilastaffetta@gmail.com (M.I.); andreamarianelli93@gmail.com (A.M.)
[5] Department of Chemistry and Technologies of Drug, Sapienza University, P.le Aldo Moro 5, 00185 Rome, Italy
* Correspondence: stefania.garzoli@uniroma1.it
† These authors contributed equally to this work.

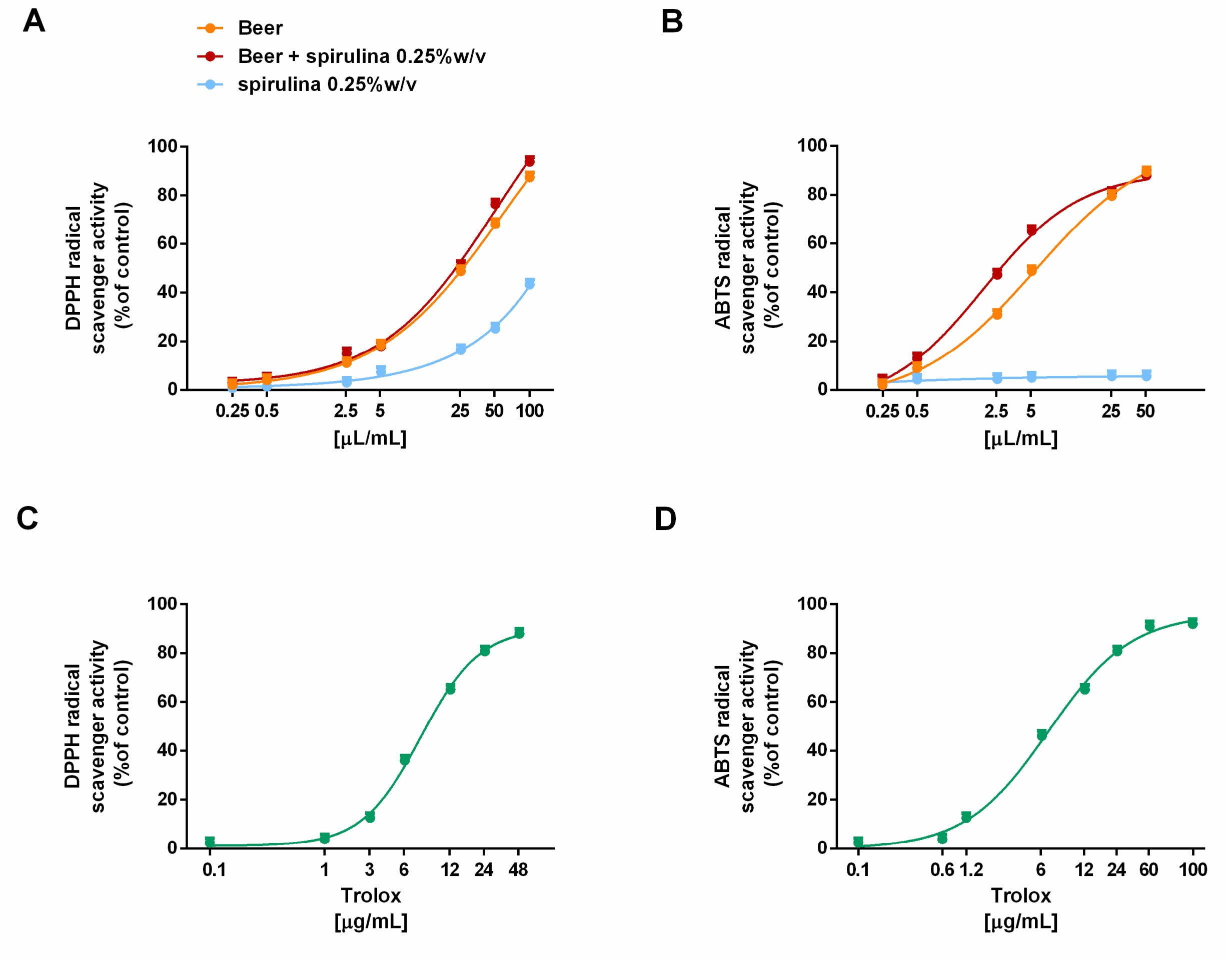

Figure S1. Radical scavenger activity of beer,beer + spirulina 0.25%w/v and spirulina at the corresponding concentrations in the beer (A,B) and of trolox (C,D) towards DPPH and ABTS radicals, respectively. Data are expressed as the average ± standard error of at least three experiments with three replicates (*n*=9).
